# Non-Ossifying Fibromas: A 2025 Review

**DOI:** 10.3390/jcm14186428

**Published:** 2025-09-12

**Authors:** Kyle Walker, Jimmy B. Smith, Niket Todi, Danielle Brown, Robert L. Randall

**Affiliations:** Department of Orthopaedic Surgery, UC Davis Health, Sacramento, CA 95817, USA

**Keywords:** non-ossifying fibroma, benign bone lesion, Pediatric Orthopedics

## Abstract

In this comprehensive review, we explore the full spectrum of the most common incidentally found bone lesions in children and adolescents. Non-ossifying fibromas (NOFs) are benign, self-limiting bone lesions that represent a significant proportion of skeletal abnormalities in this population. Beginning with their first description by Sontag and Pyle and the subsequent histological characterization by Jaffe and Lichtenstein, we trace the historical evolution of understanding NOFs, including early theories on etiology, while outlining key epidemiologic, histopathologic, and advanced imaging findings. Furthermore, we discuss diagnostic criteria, management strategies, and emerging molecular insights for NOFs, emphasizing their clinical significance. By integrating historical perspectives, current diagnostic protocols, and emerging discoveries, this paper seeks to assist clinicians in optimizing diagnostic and treatment protocols to prevent unnecessary interventions through a comprehensive understanding of NOFs.

## 1. Introduction

Did you know that the addition of fluoride to drinking water by city municipalities in the early 20th century led to the characterization of the most common bone tumor in children? The only reference to this interesting factoid was found in a single hard copy of a journal in an East Coast medical school, spurring the authors to refresh the pediatric medical community on this very common and often overanalyzed condition [1]. The incidental discovery of a bone lesion on imaging in a child is a common clinical scenario that frequently causes anxiety for patients, families, and referring physicians [2]. The primary challenge lies in differentiating benign, self-limiting entities from aggressive or malignant pathologies that require urgent intervention. Non-ossifying fibromas represent the most common of these benign lesions. While NOFs are well-known, recent discoveries in their molecular pathogenesis, evolving risk-stratification for pathologic fractures, and controversies in management warrant a contemporary and comprehensive review. This article will synthesize the historical understanding of NOFs with modern molecular insights, critically evaluate diagnostic and management algorithms, and identify key areas for future research.

Non-ossifying fibromas (NOFs) are benign fibrous bone lesions that are among the most common skeletal abnormalities observed in children and adolescents. Historically, they were known as fibrous cortical defect or metaphyseal fibrous defect. This came from an original thought that these were on the spectrum of “chronic fibrous osteomyelitis” [3]. Described in a paper by Phemister in 1929, it was noted as incidentally found fibrous lesions that were asymptomatic. They were thought to be in the spectrum of chronic osteomyelitis. Phemister speculated, “it is impossible to state when the lesion had developed, but it was apparently in a quiescent state and had it been left alone, would probably no disturbance in the future” [3]. Cultures of these lesions were found to be negative, but it was speculated that they were formed of “organisms of low virulence belonging to the pyogenic group” [3]. The consensus over the 11 cases discussed in the article was that it was likely streptococcus viridans and reflected osteomyelitis. This nomenclature has persisted in radiology reports and clinical literature. The term fibroxanthoma is also an outdated term that is now discouraged from the literature [4,5]. In the 2020 WHO update, the terms metaphyseal fibrous defect and fibrous cortical defect were no longer recommended, and the term non-ossifying fibroma was encouraged [5].

First formally described by Sontag and Pyle in 1941 as “pseudocysts”, these lesions were identified through radiographic observations in a longitudinal skeletal growth study as mentioned above [1]. Their work was particularly significant due to the scale of their study, which was one of the first to comprehensively track skeletal development over time, and their novel use of radiographic technology to identify such lesions systematically. They observed an incidence of 53% in boys and 22% in girls. Later, Jaffe and Lichtenstein (1942) applied the term “nonosteogenic fibroma of bone” and provided a detailed histological and radiographic description, emphasizing the characteristic spindle-shaped fibroblasts and hemosiderin deposits [6]. Their meticulous differentiation between NOFs and other bone lesions had a lasting impact on diagnostic practices, particularly when it comes to identifying benign versus malignant features in skeletal abnormalities. This historical context underscores the evolving understanding of NOFs, from early descriptive studies to modern diagnostic and management approaches. Often asymptomatic and discovered incidentally, NOFs have a characteristic radiographic appearance and a self-limiting natural history. Despite their benign nature, large or symptomatic lesions may require intervention to prevent complications such as pathologic fracture. Up to 60% of referrals to orthopedic oncology practices are for benign bone lesions, with NOFs comprising a significant proportion [6,7]. This high referral rate likely stems from the challenge of differentiating benign from malignant bone lesions in initial assessments, coupled with the frequent incidental discovery of NOFs during imaging for unrelated issues. Despite their benign nature, their distinctive radiographic appearance can mimic more serious conditions, prompting further evaluation to rule out malignancy. This article provides a comprehensive review of NOFs, with an emphasis on their clinical presentation, diagnosis, and management.

## 2. Etiology

The exact etiology of NOFs is not fully understood. Early studies, such as those by Sontag and Pyle in 1941, conjectured that these lesions might arise from an abnormal developmental process during skeletal growth. They described NOFs as “pseudocysts”, suggesting a misstep in ossification that results in fibroblastic proliferation within the metaphyseal regions of long bones. Jaffe and Lichtenstein (1942) expanded upon this by identifying hallmark histological features, including whorled connective tissue stroma and hemosiderin deposits, which they believed were indicative of a benign lesion with distinct biological behavior [6]. In 1986, Ritschl and Karnel discussed that NOFs only occur in areas where tendons insert into the perichondrium of the epiphyseal plate [8]. These metaphyseal areas where tendinous insertions lead to lesions are well known in the so-called “tug lesions”, such as the cortical desmoids of the distal medial femur from traction of the adductor magnus. While tug lesions can be noted in all ages, the existence of NOFs in pediatric patients only implies the role of the physeal plate. Other noted tendinous relationships may include the pes anserinus in the proximal tibia and the interosseous membrane in the distal tibia. However, some NOFs are not attached to the physeal plate and can originate from a non-fibrous or tendinous location. Thus, the etiology of NOFs is not clear-cut from histology and radiology alone. However, these early insights laid the groundwork for contemporary investigations into the cellular and molecular mechanisms of NOFs.

Despite the foundational work by Sontag, Pyle, Jaffe, and Lichtenstein, the specific factors driving lesion formation and regression remain unknown. In 2018, DNA sequencing demonstrated activating mutations in genes involved in the MAP-Kinase signaling pathway, such as KRAS, FGFR1, and NF1. In particular, the natural regression of NOF lesions is not fully understood but suspected to be related to a transient phenomenon or insufficient activation of the MAP-Kinase pathway to sustain continuous tumor growth [9]. The term “RASopathies” has been used to label a conglomerate of genetic conditions or syndromes, like NF1 and Noonan syndrome, driven by mutations in genes involved in the Ras/MAPK signaling pathway. For this reason, adding NOF to the family of RASopathies has been proposed [10].

The Ras/MAPK pathway is critical in normal cell growth, proliferation, and differentiation, particularly in osteogenesis. The pathway generally starts with a bioactive peptide binding to a cell surface receptor. This leads to the activation of receptor-linked tyrosine kinase. Downstream MAP (mitogen-activated protein) kinase is activated, leading to activation of a transcription factor and then cell division. This is a well-regulated step that is integrated into the cell cycle and is speculated to contribute to a restriction point in cell proliferation. It plays a complex role in osteogenesis, where increased Ras-MAPK signaling can antagonize bone formation but can also promote Runx2/Cbfa1 activity and osteogenic gene expression [11]. With the rise of Ras/MAPK targeting therapeutics, this duality is particularly important to elucidate.

In NOFs, KRAS, FGFR1, and NF1 represent activating mutations leading to constitutive, ligand-independent signaling down the MAPK pathway, which promotes fibroblastic proliferation over normal ossification. KRAS (Kirsten rat sarcoma viral oncogene homolog) is a known oncogene in a variety of neoplastic conditions, such as non-small cell lung cancer, colorectal cancer, and pancreatic cancer. In terms of benign bone tumors, KRAS mutations are found in brown tumors, a rare self-limiting lesion associated with hyperparathyroidism [9]. FGFR1 (Fibroblast growth factor receptor 1) is a receptor tyrosine kinase that receives signals from fibroblast growth factors. It is known to be critical in normal embryonic bone development as well as a key mutation in many genetic syndromes and skeletal dysplasia. However, patients with NOFs typically do not manifest clinical features of skeletal deformities, which reflects that FGFR1 is a somatic and not a germline mutation [9]. The NF1 (neurofibromatosis 1) gene encodes for neurofibromin, which is a negative regulator of the Ras protein. In contrast to the KRAS and FGFR1 mutations, the NF1 mutation appears to be germline. Patients with NOFs with the NF1 mutation also have neurofibromatosis type 1.

Spontaneous regression of NOFs has been theorized to be due to the fact that the mutations noted provide a proliferative stimulus, but not strong enough for malignant transformation or sustained growth. Thus, there is an eventual “burnout” or regression, leading to apoptosis as the bone matures and the microenvironment changes. This has not been fully elucidated, but is rather theorized, given the scarcity of reports in adults with non-ossifying fibromas. In contrast, those with NOFs due to germline mutations in NF1 may not have regression due to persistent stimulus in NF1 patients.

RASopathies represent a spectrum of syndromes that involve mutations in the RAS/MAPK pathway. They are noted for general craniofacial dysmorphology, cardiovascular abnormalities, musculoskeletal abnormalities, and cutaneous manifestations. Neurocognitive impairment and increased risk of benign and malignant tumors. Patients with NOFs do not typically have these other clinical manifestations despite the common pathway to mutations. This again proposes that these mutations are not germline but somatic and sporadic without sufficient stimulus to lead to further proliferation.

Jaffe-Campanacci syndrome reflects a true RASopathy that includes non-ossifying fibromas. The syndrome is characterized by multiple NOFs of the long bones and jaw and café-au-lait patches. Some patients are noted to have neurocognitive impairment along with cardiovascular malformations, hypogonadism, cryptorchidism, and ocular abnormalities [9]. There is discussion about whether Jaffe-Campanacci syndrome is a distinct entity from NF1 or a variant. Jaffe-Campanacci syndrome does not manifest with the neurofibromas of NF1. Both syndromes are allelic, meaning that they both have NF1 mutations with different presentations. Jaffe-Campanacci syndrome has been noted to have polyostotic NOFs that can lead to bone development issues and potential pathologic fractures. Whether this remains a separate clinical entity or a variant is highly controversial [12]. It is noted that the patients with non-syndromic NOFs had higher rates of recurrence than those with Jaffe-Campanacci syndrome [12]. This represents the strong clinical-genetic relationship between the RAS/MAPK pathway and NOFs.

## 3. Epidemiology

NOFs are most commonly diagnosed in individuals aged 5 to 20 years, with a peak incidence during adolescence when skeletal growth is most active. Historically, they are observed in up to 30–40% of children on routine radiographs, often as incidental findings due to their asymptomatic nature [13]. The true incidence of the lesions is unknown because patients are mostly asymptomatic. Thus, radiographic prevalence is estimated to be 30–40% and used as an estimate for the clinical population. However, a recent epidemiological study questions this estimate. Analyzing 6222 Japanese pediatric patients across 10 institutions, Emori et al. found a NOF prevalence of only 2.3%, substantially lower than the 30–40% traditionally cited [14].

In their seminal 1941 study, Sontag and Pyle studied serial radiographs of 200 children obtained by the Samuel S. Fels Research Institute as part of a natural history skeletal growth study. They noted that NOFs are generally around 1–3 cm in diameter and located most frequently in the metaphysis of long bones, particularly the medial condyle and posterior aspect of the femur [1]. Lesions most commonly appear in the distal femur (57 of 107 cases), proximal tibia (25 of 107 cases), and distal tibia (19 of 107 cases) [1]. This is likely the result of these physeal plates being the most active in the body, increasing the probability of somatic mutations leading to the development of NOFs. The distal femur is also proposed as a common location due to the relative weakness of the posterior cortex and the presence of multiple vascular channels [8]. These areas also represent areas of high mechanical stress. NOFs are not observed in sites lacking tendinous attachments near the peri-chondrium of epiphyseal cartilage, such as the femoral neck [13]. Goldin et al. in 2017 noted that in 68 NOFs in 60 patients, tendinous insertions were found in 93% of distal femoral lesions [8].

Sontag and Pyle also observed that NOFs are slightly more prevalent in males than in females (53% vs. 22%) and lesions persisted for about 2.5 years (29 months) [1]. They noted that the average age when they first appear is 46 months, with the maximum size reached around 50 months [1]. Male predominance (2.6% vs. 2.1%) and age-related patterns with NOF prevalence increasing with age and peaking at 14 were observed by Emori et al. [14]. While modern imaging modalities may detect more incidental lesions, due to the asymptomatic nature of most NOFs, there appears to be limited interest in large-scale studies for accurately determining the true incidence or updating existing data.

## 4. Clinical Presentation

### 4.1. Asymptomatic Patient

The most common presentation for patients with NOFs is an incidental finding in an asymptomatic patient. Often, the typical case will be a patient who underwent a trauma or has pain, and the tumor is noted incidentally on a radiograph. Patients are often then referred to pediatric orthopedic surgeons or orthopedic oncologists for further characterization of the lesions. The physician will often reassure the patient and the family and will potentially recommend surveillance of the lesion, depending on location or size. As most tumors will spontaneously regress, long-term surveillance is not often necessary. However, if the lesion remains stable for at least 6–12 months, surveillance is usually ceased. The surveillance course is dependent on the patient’s age, especially if they are close to the age of skeletal maturity, the size and location of the lesion, and the context of how the lesion was noted.

### 4.2. Symptomatic Patient

This scenario represents an uncommon presentation but has been reported in the literature [15]. These patients often complain of a dull ache or pain, especially with weight-bearing of the long bone. Patients will also complain of increasing pain with physical activity and loss of exercise endurance. They may also complain of swelling at the site of the tumor. These symptoms reflect direct compression of surrounding tissue from the weakening of the bone tissue or the presence of microfractures [16]. If the pain becomes persistent, this could reflect that the lesion is at risk for a pathologic fracture. These patients require close surveillance to avoid pathologic fracture through the lesion and may need prophylactic fixation.

### 4.3. Pathologic Fracture

A pathologic fracture is a rare complication of NOFs but represents a significant clinical event. They are often low-energy fractures that occur without significant trauma or injury [17]. Patients can complain of antecedent pain prior to fracture, but this is not universal in presentation. While it is common for a pathologic fracture to be minimally displaced, deformity and displacement can also occur.

Lesions with high fracture risk typically have more than one of the following features:

- Cortical thickness < 2 mm on CT imaging
- Lesion involving > 50% of the bone diameter on orthogonal radiographs
- Size > 4 cm in weight-bearing bones
- Presence of “Pac-Man Sign” or “syndesmosis sign” in the distal tibia
- Ritschl Stage B lesions with persistent symptoms
- Pain with weight-bearing activities

## 5. Radiographic and Advanced Imaging

### 5.1. Radiographic Features

**Figure 1:**
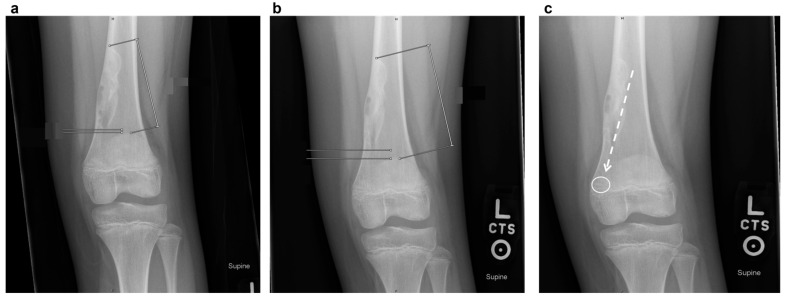
Serial radiographs demonstrating the characteristic migration pattern of NOF away from the growth plate in a 9-year-old girl over 6 months (**a**,**b**). Note the eccentric location, sclerotic rim, and “bubbly” appearance that distinguish it from other lytic lesions. Image (**c**) demonstrates the major axis of the non-ossifying fibroma with an imaginary line (dashed arrow) pointing toward the gastrocnemius insertion (white circle), which is a typical migration pattern for NOFs.

Plain radiographs typically show a well-demarcated, lytic lesion located eccentrically within the metaphysis of the affected bone. Over time, as the child grows, NOFs tend to migrate away from the growth plate towards the diaphysis due to bone remodeling [13]. Unlike enchondromas, which often present centrally within the medullary cavity and exhibit a chondroid matrix, NOFs are uniquely characterized by their eccentric location, elongated shape, fibrous matrix, and sclerotic border [18]. This differentiation underscores the diagnostic importance of radiographic positioning and lesion morphology. Table 1 summarizes some key radiographic and clinical features that distinguish NOFs from other common bone lesions. As the defect migrates away from the tendon insertion, an imaginary line can often be drawn along the lesion’s long axis, pointing toward the soft tissue insertion from which the defect presumably arises. This migration pattern is illustrated in Figure 1.

### 5.2. Staging

**Figure 2:**
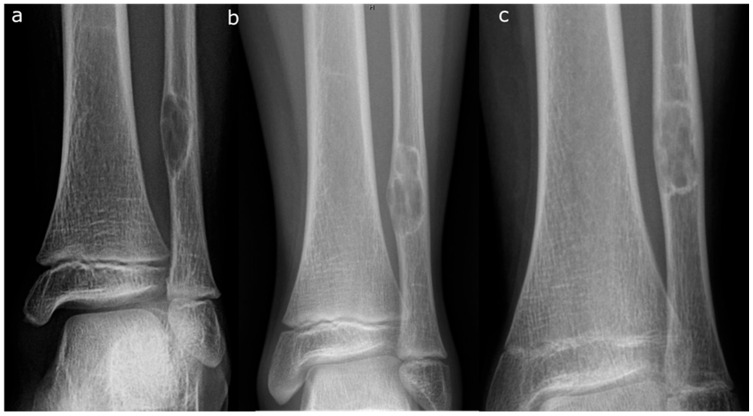
Evolution through Ritschl stages (**a**) An 8-year-old girl at the first presentation. AP radiograph showing a lytic lesion of the left distal fibula in stage A. (**b**) Two years later, the lesion was polycyclic in shape with sclerotic borders (stage B). (**c**) At the age of 12 years, there was evidence of ossification beginning at the diaphysis, representing a stage C lesion.

The most widely accepted staging system, first described by Ritschl et al. [13] and later expanded upon, is summarized below and illustrated in Figure 2.

- Stage A: Small, eccentric, cortically based lesions near the epiphysis with smooth, round borders.
- Stage B: Lesions become polycystic with clear but thin sclerotic borders, increasing in size and exhibiting variable distances from the epiphysis. The most significant growth typically occurs during this stage, transitioning from Stage A or within Stage B.
- Stage C: Lesions demonstrate increased sclerosis and reduced growth potential. The radiographic features in Stage C can be quite variable and can be a source of confusion [19].
- Stage D: Complete and homogeneous sclerosis is observed, with no further growth.

### 5.3. Advanced Imaging

Advanced imaging modalities can provide additional information in atypical cases by offering enhanced visualization of lesion characteristics and the surrounding anatomy. Routine imaging is not typically ordered in most cases and is reserved for unusual cases. For example, computed tomography (CT) is particularly useful in assessing cortical thinning and evaluating fracture risk, which can guide decisions regarding the necessity of activity restrictions or surgical intervention. Magnetic resonance imaging (MRI), with its superior soft tissue contrast, is most indicated in cases when differentiating NOFs from other pathologies, such as fibrous dysplasia or malignancy, is challenging. The decision to pursue advanced imaging often depends on the lesion’s size, location, and the presence of symptoms, as these factors significantly influence management strategies. In some cases, advanced imaging findings, like the “Pac-Man sign,” described below, may prompt closer surveillance or preemptive treatment to mitigate fracture risk. The “Syndesmosis sign” is positive when the syndesmosis can be seen inserting into a distal tibia lesion. While both signs are better seen on MRI, they can also be visualized on CT in some cases. Clinicians could benefit from clear guidelines on when to employ advanced imaging modalities in order to optimize patient care and minimize healthcare costs.

**Figure 3:**
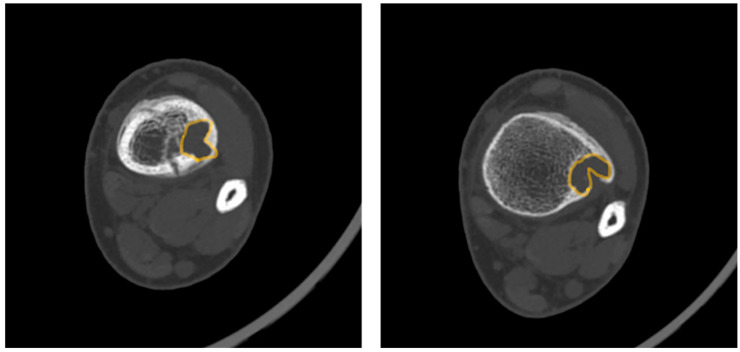
Axial computed tomography slices demonstrating the “Pac-Man Sign” in a 13-year-old boy with a pathologic fracture through a distal tibia NOF. The characteristic shape is highlighted in yellow. The “Pac-Man Sign” is a highly specific (95%) predictor of pathologic fracture, and when present with other high-risk features, prophylactic surgical intervention should be considered.

- Magnetic Resonance Imaging (MRI): MRI can help differentiate NOFs from other lesions such as fibrous dysplasia or malignancies [20]. On MRI, NOFs are typically T1-hypointense and T2-hyperintense. A peripheral, low-signal rim on all sequences corresponds to the sclerotic border seen on radiographs. Post-contrast sequences usually show minimal, peripheral enhancement, whereas more aggressive lesions often demonstrate avid, diffuse enhancement [21,22]. In 2021, Baghdadi et al. identified advanced imaging features, such as the “Pac-Man sign” and the “syndesmosis sign”, that may indicate increased fracture risk in distal tibia NOFs [23].
- Pac-Man Sign—Proliferation of bone anterior and posterior to the syndesmosis results in a shape that resembles the video game character “Pac-Man”. This sign was found to be highly specific (95%) but not very sensitive (47%) for predicting pathologic fracture (Figure 3).
- Syndesmosis Sign—Advanced imaging shows the syndesmosis inserting into the distal tibia lesion. This sign was found to be highly sensitive (94%) but less specific (48%) for predicting fracture risk.
- Computed Tomography (CT): CT can be useful for evaluating the degree of cortical thinning, which is important when assessing fracture risk [24]. CT is superior for delineating the precise cortical integrity. It allows for quantitative measurement of the cross-sectional area occupied by the lesion, which is a key factor in biomechanical models predicting fracture risk. The signs described above can also be visualized on a CT scan. Figure 3 demonstrates a “Pac-Man Sign” on CT of a 13-year-old boy who had a fracture through an NOF.
- Bone Scintigraphy: Demonstrates mild uptake, reflecting the lesion’s low metabolic activity [25].

## 6. Pathology

**Figure 4:**
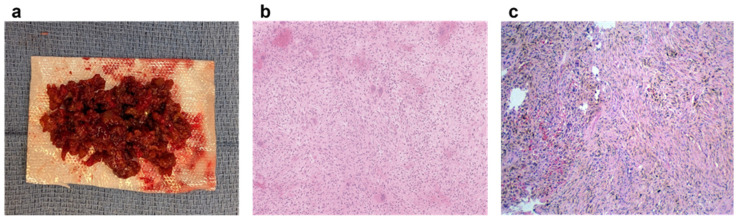
Key histologic differentiators. Gross specimen (**a**) of a non-ossifying fibroma which has been curetted out of a patient. Representative histopathology showing multi-nucleated giant cells (**b**) and hemosiderin (**c**), with both examples demonstrating a characteristic whorled stromal pattern.

On gross examination, the curetted tissue is typically rubbery and firm, with a characteristic brownish-yellow or reddish-brown color due to the high content of hemosiderin-laden macrophages. Histologically, NOFs consist of spindle-shaped fibroblasts arranged in a whorled or storiform pattern, a hallmark feature first noted by Jaffe and Lichtenstein in 1942 [6]. This distinctive arrangement, along with the absence of cellular atypia or mitotic figures, supports the benign nature of NOFs. Additionally, the presence of hemosiderin deposits and scattered multinucleated giant cells further underscores their unique histological profile, differentiating them from other bone lesions such as fibrous dysplasia or malignant tumors [26]. Jaffe and Lichtenstein also observed that these lesions lack bone formation [6], a finding that further distinguishes NOFs from other bony pathologies. The hemosiderin accounts for the brownish coloration observed grossly. Variability in cellularity, vascularity, and hemosiderin content may be observed, depending on the lesion’s stage [19]. Figure 4 demonstrates gross pathology and histology of a curetted lesion.

While diagnosis is nearly always made on standard H&E staining, in challenging cases, IHC can be used to rule out other spindle cell lesions. NOF cells are typically negative for markers like S100 and desmin [27]. Given the presence of multinucleated giant cells, H3.3G34W stains can be used to differentiate from giant cell tumors of bone. This is a stain against the G34W mutation in histone 3F3A, and mutations in the G34 position are present in 96% of giant cell tumors of the bone [28]. This mutation is not expressed in NOFs or other giant cell-rich histologic mimics.

## 7. Non-Operative Management

### 7.1. Observation and Monitoring

Most NOFs are asymptomatic and typically do not require intervention, as they resolve spontaneously during skeletal maturation. Observation is sufficient in these cases because NOFs are self-limiting and rarely lead to complications. Periodic radiographic monitoring is typically recommended to assess lesion size and ensure resolution or stabilization over time. Most lesions spontaneously regress as the individual reaches skeletal maturity [7,13]. In a retrospective review of over 1300 NOFs, Patel and Damron found that fewer than 3.5% of patients fractured or required surgery, suggesting that serial surveillance may be unnecessary for small, asymptomatic lesions [29]. Historically, large asymptomatic lesions prompted frequent radiographic surveillance and activity modification. This retrospective study challenges that intensive surveillance may represent unnecessary use of resources and radiation exposure in many cases.

### 7.2. Activity Modification

In cases of lesions presenting a higher fracture risk, activity restriction may be advised. High-impact activities, such as contact sports, are generally discouraged until the lesion stabilizes beyond Stage B [30].

### 7.3. Closed Reduction and Casting of Pathologic Fractures

In 1997, Easley et al. examined the necessity of prophylactic treatment of impending pathologic fractures in NOFs [31]. At that time, the approach supported by the orthopedic community was to treat NOFs prophylactically with curettage and bone grafting if they demonstrated more than 50% cortical involvement on AP and lateral radiographs [32]. The authors noted that many large NOFs were found incidentally and did not ultimately fracture before self-resolving. They used this clinical experience to question the status quo of the era. In their investigation, the authors observed that nearly 60% of patients with “large” NOFs did not go on to develop a fracture. While 9 out of 22 patients did sustain a fracture, all were traumatic in nature, occurred without prodromal pain, and 4 fractures occurred distant to the lesion within the same long bone. All fractures were successfully treated with closed reduction and cast immobilization.

## 8. Operative Intervention

### 8.1. Indications

Surgical intervention is reserved for symptomatic lesions, those causing significant pain, or lesions at high risk for fracture based on size and location. Factors such as cortical thinning (<1–2 mm), involvement of more than 50% of the bone diameter on two orthogonal views, lesion size (greater than 3–4 cm in major weight-bearing bones), presence of Pac-Man sign in the distal tibia, and advanced lesion staging (e.g., Ritschl Stage B) inform the decision for surgery [24,30].

### 8.2. Techniques

**Figure 5:**
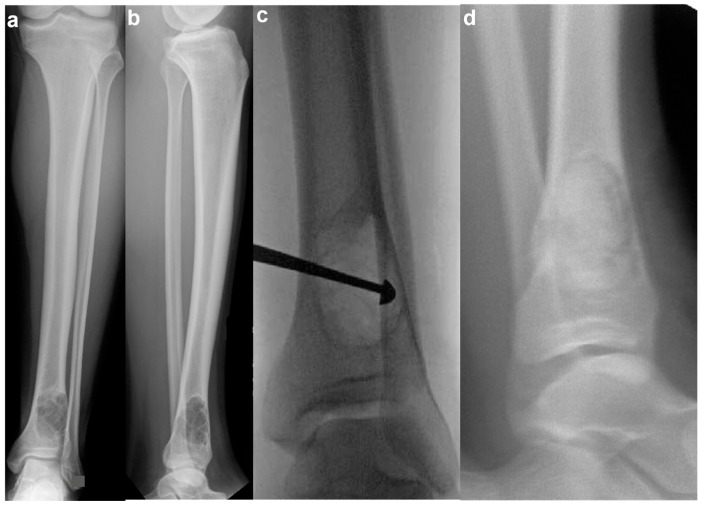
Surgical management of a symptomatic NOF. Pre-operative AP (**a**) and lateral (**b**) radiographs of a 17-year-old boy with a large, symptomatic left distal tibia NOF. Intra-operative fluoroscopy (**c**) demonstrating an AP radiograph with a curette in place. Post-operative lateral radiograph (**d**) demonstrating bone grafting in place.

- Curettage and Bone Grafting: This is by far the most performed procedure. After curettage, the cavity is filled with autograft, allograft, or synthetic bone substitute [32]. While autograft is theoretically biologically superior, the donor site morbidity makes allograft the most used graft choice. In addition, the relatively high success rate and low risk of recurrence make the risks of an additional incision with autograft prohibitive. Synthetic substitutes such as calcium sulfate and calcium phosphate can provide structural support to allow earlier weight bearing. Again, the risks of cementation, in the setting of a benign disease with good operative results, make this a less common choice. Adjuvants are typically not used in the curettage stage for NOFs, as they are in giant cell tumors of bone or aneurysmal bone cysts. NOFs are not locally aggressive, and the use of phenol or argon beam coagulation is not typically indicated to reduce recurrence. Phenol and argon have a risk of local tissue damage, making them uncommonly used in the setting of NOFs. Figure 5 demonstrates pre-, intra-, and post-operative radiographic images of a 17-year-old boy with a large symptomatic distal tibia NOF who underwent a curettage and bone grafting procedure.
- Internal Fixation: For structural support in large lesions or fractures, internal fixation with plates or intramedullary nails may be necessary.

### 8.3. Outcomes

Surgical treatment typically results in excellent outcomes, with low recurrence rates, cited as less than 5% [33]. Complications are rare but may include infection, graft resorption, or refracture [33]. Andreacchio et al. (2018) reported favorable results in surgically treated patients, particularly those with symptomatic or high-risk lesions [33]. In this case series, 9 patients with symptomatic NOFs of the lower extremity were treated with curettage, phenol treatment, and bone grafting with calcium sulfate pellets. All but one patient in this series had a Ritschl Type B lesion, and there were no long-term complications or sequelae related to the lesion or operative intervention [32,33]. Recurrence after surgical intervention is always due to incomplete curettage of the lesion.

## 9. Future Directions

Since NOFs are benign and often do not require intervention, research in this area is limited. However, further studies could focus on their origins and management. While the role of the MAPK pathway is well established, the upstream triggers for these somatic mutations remain unknown. Why do they occur in specific locations and at particular ages? Further understanding of the MAPK pathway may also reveal its involvement in other diseases. Although the risk of pathologic fractures is low, there can be serious consequences when they occur. Future research should focus on refining fracture risk prediction. While size criteria exist, the development of patient-specific biomechanical models using Finite Element Analysis (FEA) based on CT data could provide a more accurate, individualized risk assessment that could be applied broadly. For syndromic cases with multiple, problematic lesions (Jaffe-Campanacci), could MAPK inhibitors one day play a role? This remains a speculative but logical area for future investigation.

Currently, there is a limited role for non-invasive or ablative therapies when treating NOFs. However, emerging non-invasive therapies may expand treatment options for selected NOF cases. MRI-guided high-intensity focused ultrasound (MRgFUS), which has been proven effective for osteoid osteomas and painful bone metastases, could theoretically provide non-invasive alternatives for symptomatic NOFs [34,35,36]. However, no studies have specifically assessed MRgFUS for NOFs, and the typically asymptomatic nature and spontaneous regression of these lesions may limit the applicability of this technology. Future research should explore whether such interventions are warranted given the benign natural course of NOFs.

## 10. Conclusions

Non-ossifying fibromas have evolved from being viewed as a simple developmental “defect” to being recognized as a true, genetically driven benign neoplasm of the RAS/MAPK pathway. These are benign, self-limiting bone lesions most commonly occurring in children and adolescents. For the clinician, its classic radiographic features remain the key to diagnosis, allowing for confident observation in the vast majority of cases and preventing unnecessary interventions. The main challenge is identifying the small subset of lesions at high risk for pathologic fractures. While surgical treatment is highly effective when indicated, future efforts should focus on refining our risk stratification models to further optimize patient care. The story of the NOF exemplifies how molecular discoveries continue to reshape our understanding of even the most common orthopedic pathologies.

## Figures and Tables

**Table 1:** Differential diagnosis of common bone lesions that can be compared to NOFs.

Lesion	Typical Age	Location	Radiographic Features	Histology Key Feature
**Non-ossifying Fibroma**	5–20 years	Metaphysis (eccentric)	Lytic, scalloped, sclerotic rim, bubbly appearance	Storiform spindle cells, hemosiderin
**Fibrous** **Dysplasia**	<30 years	Metaphysis/Diaphysis (central)	Ground-glass, “Rind” sign, Shepherd’s Crook deformity	“Chinese character” woven bone
**Aneurysmal Bone Cyst**	<20 years	Metaphysis (central)	Expansile, lytic, fluid-fluid levels on MRI	Blood-filled spaces, giant cells
**Simple Bone Cyst (UBC)**	<20 years	Metaphysis (central)	Lytic, well-defined, “fallen leaf” sign after fracture	Thin fibrous lining, clear fluid
**Giant Cell** **Tumor**	20–40 years	Epiphysis (abuts joint)	Lytic, expansile, non-sclerotic margin	Numerous osteoclast-like giant cells

## Data Availability

This review article synthesizes existing literature and does not create or analyze new data. Data sharing is not applicable to this review article.

## Abbreviations

The following abbreviations are used in this manuscript:

NOFs	Non-ossifying fibromas
CT	Computed tomography
MRI	Magnetic Resonance Imaging

## References

[1] Sontag L., Pyle S. (1941). The Appearance and Nature of Cyst-like Areas in the Distal Femoral Metaphyses of Children. Am. J. Roentgenol..

[2] Dumitriu D., Menten R., Clapuyt P. (2014). Pitfalls in the Diagnosis of Common Benign Bone Tumours in Children. Insights Imaging.

[3] Phemister D.B. (1929). Chronic Fibrous Osteomyelitis. Ann. Surg..

[4] Merrow C., Hariharan S. (2018). Fibroxanthoma. Imaging in Pediatrics.

[5] WHO Classification of Tumours Editorial Board (2020). WHO Classification of Tumours. Soft Tissue and Bone Tumours.

[6] Jaffe H.L., Lichtenstein L. (1942). Non-Osteogenic Fibroma of Bone. Am. J. Pathol..

[7] Collier C.D., Nelson G.B., Conry K.T., Kosmas C., Getty P.J., Liu R.W. (2021). The Natural History of Benign Bone Tumors of the Extremities in Asymptomatic Children: A Longitudinal Radiographic Study. J. Bone Jt. Surg..

[8] Goldin A., Muzykewicz D.A., Dwek J., Mubarak S.J. (2017). The Aetiology of the Non-Ossifying Fibroma of the Distal Femur and Its Relationship to the Surrounding Soft Tissues. J. Child. Orthop..

[9] Baumhoer D., Kovac M., Sperveslage J., Ameline B., Strobl A.-C., Krause A., Trautmann M., Wardelmann E., Nathrath M., Höller S. (2019). Activating Mutations in the MAP-Kinase Pathway Define Non-Ossifying Fibroma of Bone. J. Pathol..

[10] Bovée J.V., Hogendoorn P.C. (2019). Non-ossifying Fibroma: A RAS-MAPK Driven Benign Bone Neoplasm. J. Pathol..

[11] Schindeler A., Little D.G. (2006). Ras-MAPK Signaling in Osteogenic Differentiation: Friend or Foe?. J. Bone Miner. Res..

[12] Jamshidi K., Motaghi P., Bagherifard A., Eigi M., Al-Baseesee H.H., Mirzaei A. (2021). Comparison of characteristic features and local recurrence in syndromic versus non-syndromic multifocal non-ossifying fibroma. J. Orthop. Sci..

[13] Ritschl P., Karnel F., Hajek P. (1988). Fibrous Metaphyseal Defects—Determination of Their Origin and Natural History Using a Radiomorphological Study. Skelet. Radiol..

[14] Emori M., Tsuchie H., Teramoto A., Shimizu J., Mizushima E., Murahashi Y., Nagasawa H., Miyakoshi N., Yamashita T. (2022). Non-ossifying fibromas and fibrous cortical defects around the knee—An epidemiologic survey in a Japanese pediatric population. BMC Musculoskelet. Disord..

[15] Moretti V.M., Slotcavage R.L., Crawford E.A., Lackman R.D., Ogilvie C.M. (2011). Curettage and graft alleviates athletic-limiting pain in benign lytic bone lesions. Clin. Orthop. Relat. Res..

[16] Shimal A., Davies A.M., James S.L., Grimer R.J. (2010). Fatigue-type stress fractures of the lower limb associated with fibrous cortical defects/non-ossifying fibromas in the skeletally immature. Clin. Radiol..

[17] Sakamoto A., Tanaka K., Yoshida T., Iwamoto Y. (2008). Nonossifying fibroma accompanied by patho-logical fracture in a 12-year-old runner. J. Orthop. Sports Phys. Ther..

[18] Chang C., Garner H., Ahlawat S. (2022). Society of Skeletal Radiology-White Paper. Guidelines for the Diagnostic Management of Incidental Solitary Bone Lesions on CT and MRI in Adults: Bone Reporting and Data System (Bone-RADS). Skeletal. Radiol..

[19] Błaż M., Palczewski P., Swiątkowski J., Gołębiowski M. (2011). Cortical Fibrous Defects and Non-Ossifying Fibromas in Children and Young Adults: The Analysis of Radiological Features in 28 Cases and a Review of Literature. Pol. J. Radiol..

[20] Rammanohar J., Zhang C., Thahir A., Krkovic M. (2021). Imaging of Non-Ossifying Fibromas: A Case Series. Cureus.

[21] Jee W., Choe B., Kang H. (1998). Nonossifying Fibroma: Characteristics at MR Imaging with Pathologic Correlation. Radiology.

[22] Stacy G., Dixon L. (2007). Pitfalls in MR Image Interpretation Prompting Referrals to an Orthopedic Oncology Clinic. Radiographics.

[23] Baghdadi S., Nguyen J.C., Arkader A. (2021). Nonossifying Fibroma of the Distal Tibia: Predictors of Fracture and Management Algorithm. J. Pediatr. Orthop..

[24] Goldin A.N., Muzykewicz D.A., Mubarak S.J. (2020). Nonossifying Fibromas: A Computed Tomography–Based Criteria to Predict Fracture Risk. J. Pediatr. Orthop..

[25] Hod N., Levi Y., Fire G. (2007). Scintigraphic Characteristics of Non-Ossifying Fibroma in Military Recruits Undergoing Bone Scintigraphy for Suspected Stress Fractures and Lower Limb Pains. Nucl. Med. Commun..

[26] Choi J., Ro J. (2021). The 2020 WHO Classification of Tumors of Bone: An Updated Review. Adv. Anat. Pathol..

[27] Kraus M.D., Haley J.C., Ruiz R., Essary L., Moran C.A., Fletcher C.D. (2001). “Juvenile” xanthogranuloma: An immunophenotypic study with a reappraisal of histogenesis. Am. J. Dermatopathol..

[28] Yamamoto H., Iwasaki T., Yamada Y., Matsumoto Y., Otsuka H., Yoshimoto M., Kohashi K., Taguchi K., Yokoyama R., Nakashima Y. (2018). Diagnostic utility of histone H3.3 G34W, G34R, and G34V mutant-specific antibodies for giant cell tumors of bone. Hum. Pathol..

[29] Patel R.R., Damron T.A. (2024). The Role of Surveillance in Predicting Fracture in Pediatric Patients with Incidentally Discovered Nonossifying Fibromas and Fibrous Cortical Defects: Is It Worth It?. J. Pediatr. Orthop..

[30] Herget G.W., Mauer D., Krauß T., El Tayeh A., Uhl M., Südkamp N.P., Hauschild O. (2016). Non-Ossifying Fibroma: Natural History with an Emphasis on a Stage-Related Growth, Fracture Risk and the Need for Follow-Up. BMC Musculoskelet. Disord..

[31] Easley M.E., Kneisl J.S. (1997). Pathologic fractures through nonossifying fibromas: Is prophylactic treatment warranted?. J. Pediatr. Orthop..

[32] Arata M.A., Peterson H.A., Dahlin D.C. (1981). Pathological Fractures through Non-Ossifying Fibromas. Review of the Mayo Clinic Experience. J. Bone Jt. Surg..

[33] Andreacchio A., Alberghina F., Testa G., Canavese F. (2018). Surgical Treatment for Symptomatic Non-Ossifying Fibromas of the Lower Extremity with Calcium Sulfate Grafts in Skeletally Immature Patients. Eur. J. Orthop. Surg. Traumatol..

[34] Bongiovanni A., Foca F., Oboldi D., Diano D., Bazzocchi A., Fabbri L., Mercatali L., Vanni S., Maltoni M., Bianchini D. (2022). 3-T magnetic resonance-guided high-intensity focused ultrasound (3 T-MR-HIFU) for the treatment of pain from bone metastases of solid tumors. Support Care Cancer.

[35] Singh V.A., Shah S.U., Yasin N.F., Abdullah B.J.J. (2017). Magnetic resonance guided focused ultrasound for treatment of bone tumors. J. Orthop. Surg..

[36] Rodrigues D.B., Stauffer P.R., Vrba D., Hurwitz M.D. (2015). Focused ultrasound for treatment of bone tumours. Int. J. Hyperth..

